# Transient Receptor Potential Canonical Channels 4 and 5 Mediate *Escherichia coli*-Derived Thioredoxin Effects in Lipopolysaccharide-Injected Mice

**DOI:** 10.1155/2018/4904696

**Published:** 2018-06-10

**Authors:** Domingos M. S. Pereira, Saulo J. F. Mendes, Khadija Alawi, Pratish Thakore, Aisah Aubdool, Nágila C. F. Sousa, João F. R. da Silva, José A. Castro, Ione C. P. Pereira, Luís C. N. Silva, Marcos A. G. Grisotto, Valério Monteiro-Neto, Soraia K. P. Costa, Robson da Costa, João B. Calixto, Susan D. Brain, Elizabeth S. Fernandes

**Affiliations:** ^1^Programa de Pós-graduação, Universidade Ceuma, Rua dos Castanheiros, no 1, Renascença II, São Luís, MA, Brazil; ^2^Vascular Biology and Inflammation Section, BHF Cardiovascular Centre of Excellence, King's College London, Waterloo Campus, London, UK; ^3^Centro de Ciências da Saúde, Universidade Federal do Maranhão, São Luís, MA, Brazil; ^4^Departamento de Farmacologia, Universidade de São Paulo, Av. Prof. Lineu Prestes, Butantan, SP, Brazil; ^5^Wolfson Centre for Age-Related Diseases, King's College London, London Bridge, London, UK; ^6^Centro de Inovação e Ensaios Pré-Clínicos - CIEnP, Av. Luiz Boiteux Piazza, no 1302 - Cachoeira do Bom Jesus, Florianópolis, SC, Brazil

## Abstract

Thioredoxin plays an essential role in bacterial antioxidant machinery and virulence; however, its regulatory actions in the host are less well understood. Reduced human Trx activates transient receptor potential canonical 5 (TRPC5) in inflammation, but there is no evidence of whether these receptors mediate bacterial thioredoxin effects in the host. Importantly, TRPC5 can form functional complexes with other subunits such as TRPC4. Herein, *E. coli*-derived thioredoxin induced mortality in lipopolysaccharide- (LPS-) injected mice, accompanied by reduction of leukocyte accumulation, regulation of cytokine release into the peritoneum, and impairment of peritoneal macrophage-mediated phagocytosis. Dual TRPC4/TRPC5 blockade by ML204 increased mortality and hypothermia in thioredoxin-treated LPS mice but preserved macrophage's ability to phagocytose. TRPC5 deletion did not alter body temperature but promoted additional accumulation of peritoneal leukocytes and inflammatory mediator release in thioredoxin-administered LPS mice. Thioredoxin diminished macrophage-mediated phagocytosis in wild-type but not TRPC5 knockout animals. TRPC5 ablation did not affect LPS-induced responses. However, ML204 caused mortality associated with exacerbated hypothermia and decreased peritoneal leukocyte numbers and cytokines in LPS-injected mice. These results suggest that bacterial thioredoxin effects under LPS stimuli are mediated by TRPC4 and TRPC5, shedding light on the additional mechanisms of bacterial virulence and on the pathophysiological roles of these receptors.

## 1. Introduction

Thioredoxin (Trx) is a redox protein produced by all species, from bacteria to humans. Trx plays a pivotal role as an antioxidant molecule; however, its immunomodulatory actions and subsequent role in infections are not well understood and may be species dependent. Indeed, bacteria-derived Trx has been linked to increased bacterial virulence [1], whilst host-derived Trx has been associated with bacteria evasion from the host's immune system [2].

Mammalian Trx was previously shown to regulate a series of intracellular cascades including activation of gene transcription and induction of apoptosis [3–6]. Data obtained from *in vitro* experiments suggest that once released, the mammalian Trx acts extracellularly, modulating cytokine production and as a chemoattractant for monocytes and neutrophils [7]. Interestingly, Trx overexpressing mice are protected from lipopolysaccharide- (LPS-) induced hepatic damage by presenting reduced cytocrome c-mediated apoptosis [8]. Also, Trx expression is found to be upregulated in different organs (heart, lungs, and liver) in LPS-treated mice [9]. These evidences suggest a potential protective effect for host Trx in sepsis. More recently, Ma et al. [2] showed that *Streptococcus* sp. uses host-produced Trx to evade phagocytosis. To add another layer of complexity to Trx effects, its production and release by bacteria such as *Salmonella* sp. increases bacterial virulence in infected mice [1]. This is suggested to be due to Trx antioxidant properties [10–13]. Bacterial Trx was also shown to be crucial to the increased mortality observed in *Salmonella*-induced infection *in vivo* [14].

In its reduced form, human Trx was found to activate the transient receptor potential canonical 5 (TRPC5) subunits [15], a receptor found on sensory neurones and nonneuronal cells such as endothelial and kidney cells. The ability of TRPC5 to regulate the inflammatory response was previously suggested [15, 16]; however, its role in sepsis is yet to be addressed. Also, it is unclear whether bacterial Trx immunomodulatory actions in the host depend on TRPC5 activation. Importantly, TRPC5 can form functional complexes with other receptors of the same family, such as TRPC4 [15, 17], which was recently suggested to become upregulated under LPS stimuli [18, 19].

Therefore, we investigated the contribution of TRPC5 and TRPC4 channels to the systemic inflammatory response (SIRS) caused by LPS by using TRPC5 knockout (TRPC5^−/−^) and wild-type (TRPC5^+/+^) mice and a TRPC4/TRPC5 antagonist. We further assessed the contribution of these receptors to bacterial Trx-induced responses in LPS-injected mice. We suggest that the effects of *Escherichia coli*-derived Trx in LPS-induced responses depend on the activation of both TRPC4 and TRPC5 channels.

## 2. Materials and Methods

### 2.1. Mice

Nonfasted male C57BL/6 and 129Si/SvImJ TRPC5^+/+^ and TRPC5^−/−^ mice (2-3 months of age) were used. C57BL/6 animals were obtained from the animal's facility of the Universidade Ceuma (UNICEUMA). TRPC5^+/+^ and TRPC5^−/−^ breeding pairs were bred at King's College London (KCL) Biological Service Unit from mice provided by Prof. D.E. Clapham (Howard Hughes Medical Institute, Boston, U.S.A) [20]. Animals were housed in a climatically controlled environment (room temperature of 22 ± 2°C) and humidity of around 60%, on a 12–12 h light/dark cycle (lights on at 07:00), with free access to water and food. All experiments were conducted under the guidelines of the United Kingdom Home Office Animals (Scientific Procedures) Act 1986 and in accordance with the Brazilian Society for Animal Welfare (SBCAL), following approval by the KCL Animal Care and Ethics Committees and the Ethics Committee of UNICEUMA, respectively. All experiments were conducted in a blinded manner. Animals were randomly assigned into groups and the experimenter was blinded towards the treatment and the genetic background of animals during the experiment.

### 2.2. Pharmacological Treatments

C57BL/6, TRPC5^+/+^, and TRPC5^−/−^ mice received a subcutaneous (s.c.) injection of phosphate-buffered saline (PBS; Sigma-Aldrich) containing bacterial Trx (20 *μ*g/150 *μ*l/animal, twice a day; from *E. coli*; Sigma-Aldrich) for 3 days prior to the induction of SIRS. In order to assess the role of TRPC4 and TRPC5 complexes in LPS-induced responses, C57BL/6 mice received ML204 [16, 21] (1 mg/kg, 150 *μ*l/animal, twice a day; Sigma-Aldrich) for 5 days and then LPS. In a separate set of experiments, C57BL/6 animals received ML204 (1 mg/kg, twice a day; in 6% dimethyl sulfoxide (DMSO) in PBS) for 2 days alone, and then, this drug was coinjected with bacterial Trx (20 *μ*g/animal, twice a day) for another 3 days prior to LPS challenge. Vehicle-treated mice were used as controls.

### 2.3. Induction of SIRS

Animals received an i.p. injection of saline (0.9%) containing LPS (11.25 million of EU/kg; obtained from *E. coli* serotype O111:B4; Sigma-Aldrich) [22], and the SIRS was allowed to develop for 24 h. Vehicle-treated mice were used as controls. Baseline body weights and temperature were registered prior (baseline) and 24 h after LPS injection and the results are expressed as percentage (%) of body weight and temperature in relation to baseline. The severity of SIRS (denoted by changes in grooming behaviour and mobility and presence of piloerection and weeping eyes) was evaluated at 24 h following LPS injection, as previously described [22]. In a separate series of experiments, mortality rates were evaluated over 96 h following SIRS induction in independent groups of C57BL/6 mice (8–10 mice/group).

### 2.4. AST and Creatinine Levels

Dysfunction of the heart/liver and kidneys was assessed by measuring aspartate aminotransferase (AST) and creatinine levels, respectively, in plasma samples of mice 24 h after the administration of LPS or vehicle by using commercial kits, according with the manufacturer's instructions (Sigma-Aldrich). For this, blood samples were obtained by cardiac puncture in animals previously anaesthetized. The plasma was then obtained by centrifugation of heparinised blood samples at 1500 rpm for 20 min. Results are expressed as milliunits/ml (AST) and ng/*μ*l (creatinine).

### 2.5. Collection of Peritoneal Lavage Samples and Cell Counts

Twenty-four hours following SIRS induction, the animals were anaesthetized, and then the peritoneal lavage fluid (PELF) was collected by laparotomy as described by Fernandes et al. [23]. The peritoneal cavity was washed with 3 ml of sterile PBS and an aliquot was used for total and differential counts (×10^6^/ml) of peritoneal leukocytes. Another aliquot of the PELF (500 *μ*l) was separated for *in vitro* analysis of macrophage-mediated phagocytosis; the rest was centrifuged at 1500 rpm for 20 min, and the supernatant was collected and kept at −80°C until further analysis of the levels of inflammatory mediators.

### 2.6. Nitric Oxide (NO^x^) Levels

The NO_2_^−^/NO_3_^−^ content was measured by the Griess assay as an indicator of NO production in the peritoneal lavage, according to the method described by Mendes et al. [22]. For this, 80 *μ*l of sample were incubated with 20 *μ*l of 1 U/ml nitrate reductase (Sigma-Aldrich) and 10 *μ*l of 1 mM NADPH (Sigma-Aldrich) for 30 min at 37°C in a 96-well plate. Then, 100 *μ*l of Griess reagent (Sigma-Aldrich) was added and incubated for 15 min at 37°C. Absorbance at 550 nm was measured immediately using a spectrophotometer (plate reader MB-580; Heales, Shenzhen, China). After subtraction of background readings, the absorbance in each sample was compared with that obtained from a sodium nitrite (0–100 *μ*M) standard curve. Results are expressed as levels of NO^x^ in *μ*M.

### 2.7. Analysis of PELF H_2_O_2_

Hydrogen peroxide (H_2_O_2_) production by peritoneal inflammatory cells was measured by using a H_2_O_2_/peroxidase assay kit (Amplex Red H_2_O_2_/Peroxidase assay kit; Molecular Probes, Invitrogen). The assay was performed as previously described [22]. Briefly, 50 *μ*l of PELF were incubated with 50 *μ*l of a solution containing NaPO_4_ 0.05 M (pH 7.4), HRP 0.2 U/ml, and Amplex Red reagent (10-acetyl-3,7-dihydroxyphenoxazine) 25.7 mg/ml for 2 h at 37°C. Samples incubated with NaPO_4_ 0.05 M only were used as controls. After incubation, the reaction was read at 560 nm. Absorbance readings, obtained for samples incubated in the absence or presence of Amplex Red reagent, were compared with a H_2_O_2_ standard curve (0–40 *μ*M). Results are expressed as levels of H_2_O_2_ in *μ*M.

### 2.8. Cytokine Measurements

The levels of PELF cytokines (TNF*α*, IL-6, and IL-10) were evaluated by using mouse cytometric bead array (CBA) cytokine (BD Biosciences) and V-PLEX Proinflammatory Panel 1 Mouse (Meso Scale Discovery) multiplex kits according with the manufacturer's instructions. Readings were compared with those of appropriate standard curves, and results are expressed as picograms of cytokine per millilitre of PELF (pg/ml).

### 2.9. Macrophage-Mediated Phagocytosis

The peritoneal cells obtained were centrifuged (1500 rpm, 10 min, 4°C) and resuspended in DMEM Media - GlutaMAX™ (Thermo Fisher Scientific) containing 10% fetal bovine serum (*v*/*v*; Thermo Fisher Scientific) and penicillin-streptomycin (1x; Sigma-Aldrich). Cells (6 × 10^5^/well) were incubated in eight chamber culture slides (BD Falcon) at 37°C in 5% CO_2_, and after 2 h, the nonadhered cells were removed. Adherent cells (macrophages) were then incubated with 2 *μ*M fluorescent latex beads (1 : 100; 5 *μ*l/well) for 12 h. After the incubation period, the cell culture medium was removed and each well was processed and analyzed as described by Fernandes et al. [23]. Slides were analyzed by microscopy (Olympus BX51 or Zeiss Axio Z2; bright field). Images were acquired by an Olympus color view 3 or Zeiss AxioCam ICc5 camera and visualized in Cell P or ZEN programmes. Two lots of 100 cells were counted for each sample, and the average for each sample was considered as an *n* number. Results are expressed as number of phagocytosed beads per 100 cells.

### 2.10. Statistical Analysis

The results are presented as the mean ± standard error (SE). The percentage of inhibition is reported as the mean ± SE for each individual experiment. Statistical comparison was performed by analysis of variance followed by the Bonferroni test. The results of the severity score analysis are expressed as the median (minimum-maximum) values and were analyzed using Kruskal-Wallis test followed by Dunn's test for multiple comparisons. Survival curves were analyzed by the nonparametric Mantel-Cox test. *p* < 0.05 was considered significant.

## 3. Results

### 3.1. Dual Blockade of TRPC4 and TRPC5 Induces Mortality Associated with Increased Hypothermia in Mice with LPS-Induced SIRS

**Figure 1:**
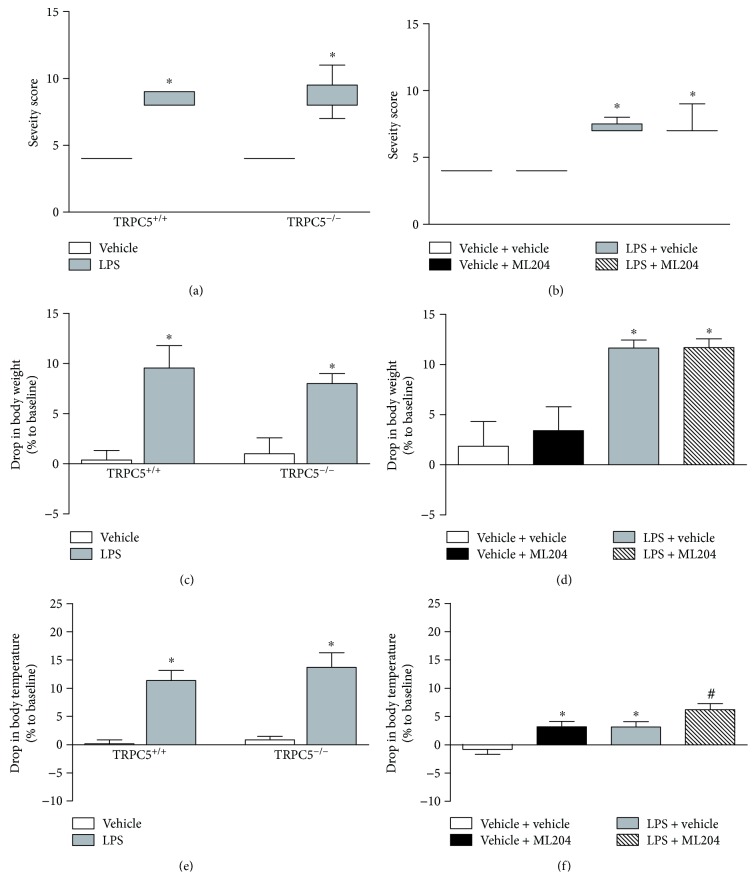
Effects of TRPC5 deletion and TRPC4/TRPC5 antagonism in the severity, body temperature, and weight of SIRS mice. (a) Severity of SIRS, (c) drop in body weight, and (e) temperature in TRPC5^+/+^ and TRPC5^−/−^ mice injected with vehicle (PBS; *n* = 4–8) or lipopolysaccharide (LPS; *n* = 7–14). (b) Severity of SIRS, (d) drop in body weight, and (f) temperature in mice treated with vehicle (6% DMSO in PBS; *n* = 8–16) or the TRPC4/TRPC5 antagonist ML204 (1 mg/kg; *n* = 8–16) subcutaneously, twice a day, for 5 days prior to LPS injection. Non-SIRS (PBS-injected) mice were used as controls (*n* = 5–8). ^∗^*p* < 0.05 differs from vehicle-injected controls; ^#^*p* < 0.05 differs from LPS-injected controls.

Herein, LPS-injection was used as a model of SIRS associated with gram-negative bacteria. We initially assessed the contribution of TRPC5 and TRPC4 channel activation to LPS-induced SIRS; therefore, systemic alterations such as body weight and temperature, markers of organ damage, severity of disease, and mortality rate were evaluated. As expected, mice injected with LPS exhibited severe disease which was accompanied by a marked drop in body weight and temperature in comparison with the control group, irrespective of genotype (Figure 1(a), 1(c), and 1(e), respectively; *p* < 0.05). Similarly, repeated pretreatment with the dual TRPC4/TRPC5 blocker ML204 did not alter SIRS severity or body weight in LPS mice (Figure 1(b) and 1(d)). On the other hand, hypothermia was more pronounced (twofold increase) in SIRS mice administered with ML204 in comparison with their vehicle controls (Figure 1(f); *p* < 0.05).

**Figure 2:**
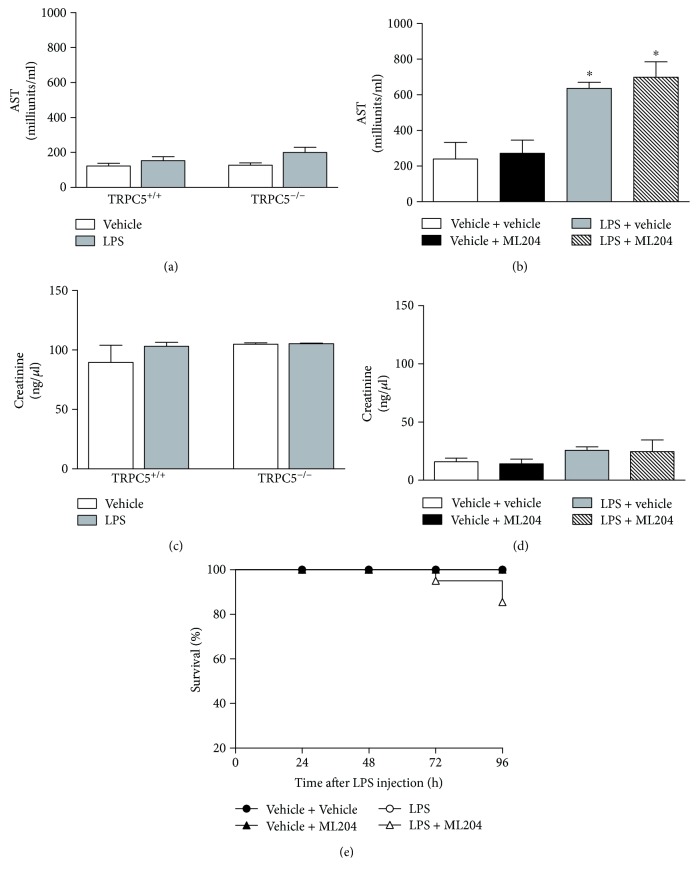
Effects of TRPC5 deletion and TRPC4/TRPC5 antagonism in organ failure and survival of SIRS mice. Circulating (a) aspartate aminotransferase (AST) and (c) creatinine levels in TRPC5^+/+^ and TRPC5^−/−^ mice injected with vehicle (PBS; *n* = 4) or lipopolysaccharide (LPS; *n* = 7). Circulating levels of (b) AST and (d) creatinine in mice treated with vehicle (6% DMSO in PBS; *n* = 6) or the TRPC4/TRPC5 antagonist ML204 (1 mg/kg; *n* = 6) subcutaneously, twice a day, for 5 days prior to LPS injection. Non-SIRS (PBS-injected) mice were used as controls (*n* = 5–8). Survival rates (e) were registered in SIRS and non-SIRS mice administered with vehicle or ML204 (*n* = 10/group). ^∗^*p* < 0.05 differs from vehicle-injected controls.

Loss of TRPC5 signalling was previously suggested to attenuate liver injury caused by cholestasis [24] and to improve kidney function in LPS-injected mice [25]. Data depicted in Figure 2(a)–2(d) demonstrates that neither TRPC5 ablation nor TRPC4/TRPC5 blockade significantly altered the levels of AST and creatinine (indicators of liver and kidney damage, resp.) in mice with SIRS; still, TRPC5^−/−^ mice with SIRS exhibited higher levels of AST (1.6-fold increase) than their vehicle controls and TRPC5^+/+^ injected with LPS. Assessment of survival showed that ML204 pretreatment causes mortality (15%) in C57BL/6 mice with SIRS whilst no deaths were registered for those administered with vehicle (Figure 2(e)).

ML204 did not affect severity, body weight, or organ function (Figure 1(b) and 1(d); Figure 2(b) and 2(d)); but caused hypothermia in non-SIRS mice in comparison with vehicle controls (Figure 1(f); *p* < 0.05). Also, ML204 had no effects in the survival of the same mice (Figure 2(f)).

### 3.2. *E. coli*-Derived Trx Causes Mortality in SIRS Mice and This Is Further Exacerbated by TRPC4/TRPC5 Antagonism

**Figure 3:**
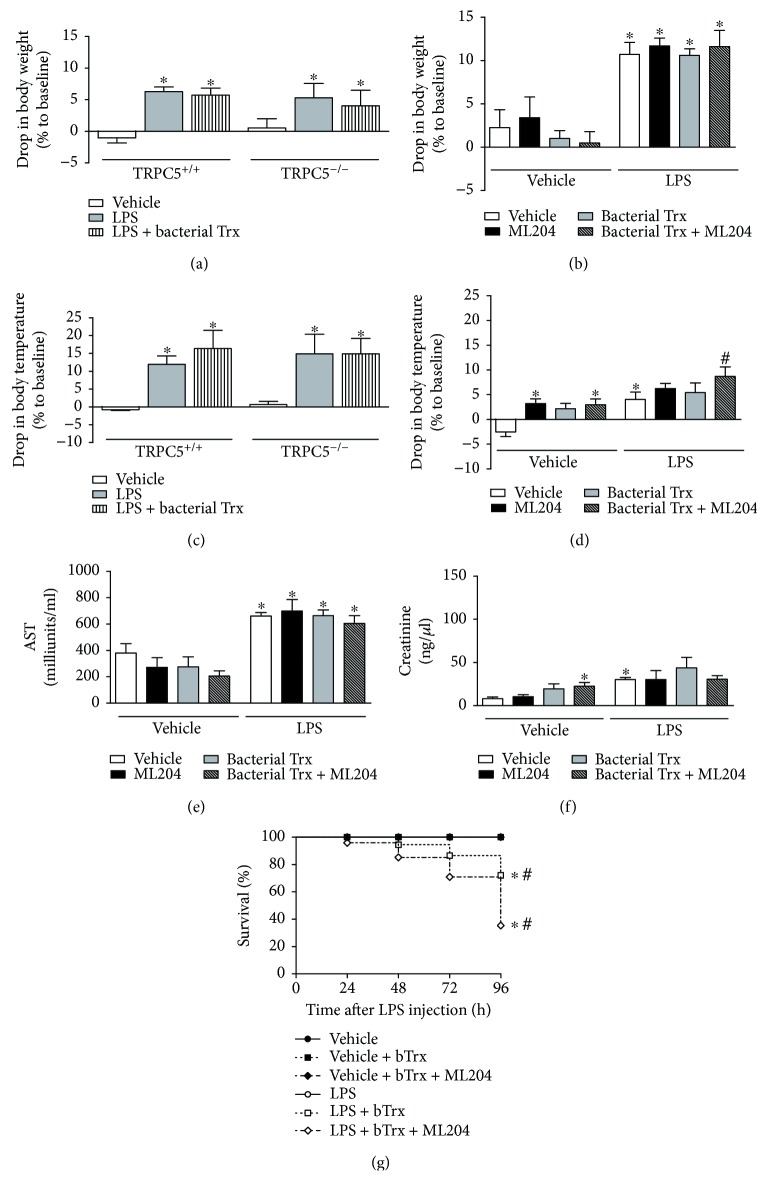
Effects of bacterial thioredoxin (Trx) in body temperature and weight, organ failure, and survival of SIRS mice. Bacterial Trx (20 *μ*g/150 *μ*l/mouse, subcutaneously, twice a day, for 3 days prior to lipopolysaccharide (LPS) injection) effects in (a) body weight and (c) temperature of TRPC5^+/+^ and TRPC5^−/−^ mice injected with vehicle (PBS; *n* = 6) or LPS (*n* = 6). Effects of bacterial Trx (20 *μ*g/150 *μ*l/mouse, subcutaneously, twice a day, for 3 days prior to LPS injection) in (b) body weight and (d) temperature, (e) AST, and (f) creatinine levels in SIRS mice treated with vehicle (6% DMSO in PBS; *n* = 6) or the TRPC4/TRPC5 antagonist ML204 (1 mg/kg; *n* = 6) subcutaneously, twice a day, for 5 days prior to LPS injection. Non-SIRS (PBS-injected) mice were used as controls (*n* = 5–8). Survival rates (e) were registered for mice administered with vehicle, ML204, Trx, or Trx + ML204 (*n* = 8/group). ^∗^*p* < 0.05 differs from vehicle-injected controls; ^#^*p* < 0.05 differs from LPS-injected controls.

We next evaluated the effects of bacterial Trx in LPS-injected mice. Treatment with this protein was not able to affect body weight or temperature in SIRS mice (Figure 3(a) and 3(c)). Similarly, ML204 did not alter body weight in SIRS mice treated with bacterial Trx; however, the same animals exhibited increased hypothermia (2.7-fold) in comparison with LPS controls (Figure 3(d); *p* < 0.05). Treatment with bacterial Trx had no effects in LPS-induced liver and kidney damage, and this was not altered by treatment with ML204 (Figure 3(e) and 3(f)). However, bacterial Trx caused marked mortality (28%) in LPS mice (Figure 3(g); *p* < 0.05), an effect that was significantly exacerbated by treatment with ML204 (mortality of 65%) (Figure 3(g); *p* < 0.05).

The administration of bacterial Trx in non-SIRS mice caused nonsignificant hypothermia and elevation of creatinine in these mice (Figure 3(d) and 3(f); *p* > 0.05), without affecting their survival or AST levels (Figure 3(e) and 3(g)). ML204 treatment did not affect Trx effects in non-SIRS mice (Figure 3).

### 3.3. Treatment with ML204 or Bacterial Trx but Not TRPC5 Ablation Reduces Peritoneal Cell Numbers in Mice

**Figure 4:**
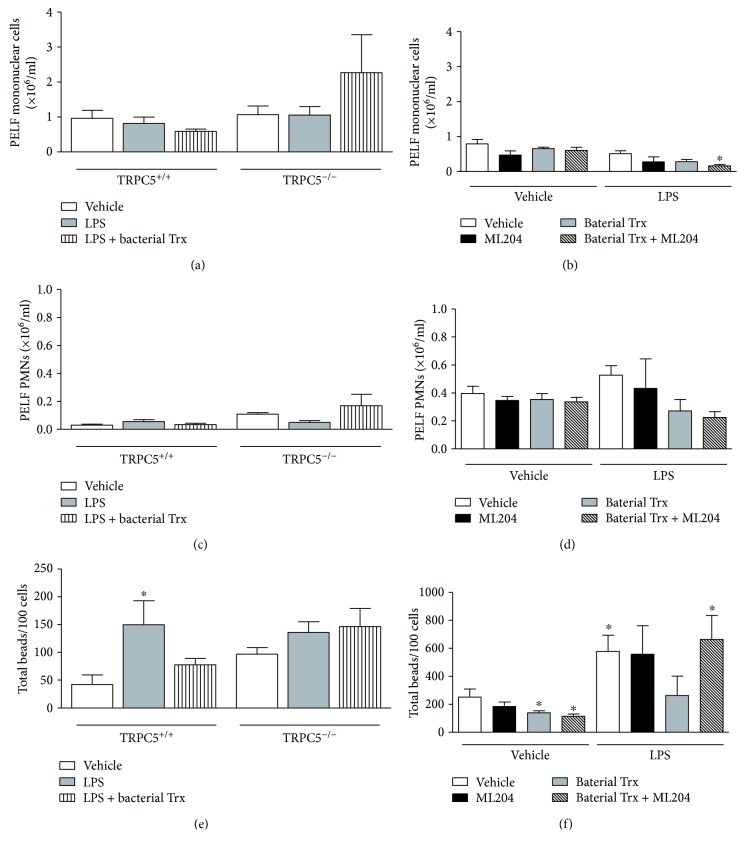
Effects of bacterial thioredoxin (Trx) in the number of peritoneal leukocytes and macrophage-mediated phagocytosis in SIRS mice. Bacterial Trx (20 *μ*g/150 *μ*l/mouse, subcutaneously, twice a day, for 3 days prior to lipopolysaccharide (LPS) injection) effects in the numbers of peritoneal (a) mononuclear and (c) polymorphonuclear (PMN) cells and (e) macrophage-mediated phagocytosis in TRPC5^+/+^ and TRPC5^−/−^ mice injected with vehicle (PBS; *n* = 4) or LPS (*n* = 6). Effects of bacterial Trx (20 *μ*g/150 *μ*l/mouse, subcutaneously, twice a day, for 3 days prior to LPS injection) in the numbers of peritoneal (b) mononuclear and (d) polymorphonuclear (PMN) cells and (f) macrophage-mediated phagocytosis in SIRS and non-SIRS mice treated with vehicle (6% DMSO in PBS; *n* = 6–9) or the TRPC4/TRPC5 antagonist ML204 (1 mg/kg; *n* = 5–9) subcutaneously, twice a day, for 5 days prior to LPS injection. ML204-treated mice were used for comparison (*n* = 5–9). ^∗^*p* < 0.05 differs from vehicle-injected controls.

In order to investigate the participation of TRPC4 and TRPC5 in the local alterations caused by the intraperitoneal injection of LPS, the PELF samples were analyzed, and the numbers of peritoneal leukocytes were counted. It was observed that TRPC5 ablation does not affect leukocyte accumulation into the peritoneum of LPS-injected mice, denoted by the numbers of mononuclear and polymorphonuclear (PMN) cells (Figure 4(a) and 4(c)). On the other hand, administration of ML204 caused reduction (46%) in the peritoneal mononuclear cell population (Figure 4(b)) of LPS-injected animals in comparison with LPS controls; however, this was not significant. A similar effect was observed for those which received bacterial Trx, as they presented lower numbers of mononuclear (44%) and PMN (48%) cells than LPS-injected controls (Figure 4(b) and 4(d)). Analysis of PELF population in non-SIRS mice showed that ML204 reduces the number of mononuclear cells (40%) (Figure 4(b)), with no effects in PMNs (Figure 4(d)).

### 3.4. TRPC5 but Not Dual TRPC4/TRPC5 Antagonism Promotes the Accumulation of Leukocytes in the Peritoneal Cavity of Trx-Treated SIRS Mice

The contribution of TRPC4/TRPC5 subunits to the leukocyte influx in the peritoneal cavity of SIRS mice injected with bacterial Trx was also assessed. Although not significant, SIRS TRPC5^−/−^ treated with bacterial Trx exhibited higher numbers of leukocytes in their peritoneal cavity than TRPC5^+/+^ mice with SIRS administered with the same protein (3.8-fold and 5-fold increase for mononuclear and PMN cells, resp.) and LPS-injected TRPC5^−/−^ mice (2.1-fold and 3.4-fold increase for mononuclear and PMN cells, resp.) (Figure 4(a) and 4(c)). Treatment with ML204 did not significantly affect the peritoneal population of Trx-administered SIRS mice (Figure 4(a) and (c)); however, it further decreased the number of mononuclear cells in these mice.

Trx had no effects in PELF leukocytes of non-SIRS mice (Figure 4(b) and 4(d)), and this was not altered by ML204 treatment (Figure 4(b) and 4(d)).

### 3.5. *E. coli*-Derived Trx Inhibits LPS-Induced Phagocytosis in Macrophages via Activation of TRPC5

Bacterial Trx facilitates evasion of phagocytosis [2].  Figure 4 shows that LPS-induced phagocytosis in macrophages was reduced by Trx treatment (~51%), an effect that was prevented by TRPC5 ablation or dual TRPC4/TRPC5 blockade (Figure 4(e) and 4(f)). Trx also reduced phagocytosis in macrophages of non-SIRS mice (45%) (Figure 4(f)); this response was not affected by ML204 treatment (Figure 4(f)).

### 3.6. TRPC4 and TRPC5 Differentially Regulate the Release of Peritoneal Inflammatory Mediators during SIRS

Changes in the generation of inflammatory mediators are essential to the host response to infection; thus, the levels of peritoneal mediators were analyzed. LPS triggered the release of cytokines, NO, and H_2_O_2_ in the peritoneal cavity of TRPC5-expressing mice (Tables 1 and 2). LPS-induced increases in cytokine and NO^x^ concentrations were higher in C57BL/6 (Table 1) than in TRPC5^+/+^ (Table 2) in comparison with their vehicle controls. On the other hand, LPS-injected TRPC5^+/+^ mice release greater amounts of H_2_O_2_ than mice of the C57BL/6 strain in comparison with vehicle controls (Tables 1 and 2). Fold increases in C57BL/6 mice were of 1.9, 191.9, 1.8, and 2.0 for TNF*α*, IL-6, IL-10, and NO^x^, respectively; whilst TRPC5^+/+^ animals presented with 1.5-, 7.3-, 1.2-, and 1.7-fold increases for TNF*α*, IL-6, IL-10, and H_2_O_2_ levels, respectively.

Whilst ML204 treatment decreased the production of cytokines (IL-6 and IL-10) in mice with SIRS (Table 2), the lack of TRPC5 did not affect this response (Table 1). Bacterial Trx had no effects in the release of inflammatory mediators in the peritoneal cavity of SIRS TRPC5^+/+^ mice (Table 1) but impaired the production of peritoneal cytokines (TNF*α*, IL-6, and IL-10) and H_2_O_2_ in the C57BL/6 strain (Table 2). Also, Trx caused increased release of TNF*α*, IL-6, NO, and H_2_O_2_ in TRPC5^−/−^ animals injected with LPS in comparison with LPS controls or TRPC5^+/+^ mice administered with this protein (Table 1). ML204 treatment partially reversed Trx-induced reduction of peritoneal IL-6 and IL-10 in SIRS mice (Table 2).

The concentrations of PELF inflammatory mediators were also evaluated in non-SIRS mice. Data depicted on Table 2 shows that when administered with ML204, these mice produce higher levels of TNF*α* (1.8-fold increase), IL-10 (2.5-fold increase), and H_2_O_2_ (1.9-fold increase) but are impaired to release NO. Similarly, non-SIRS mice treated with bacterial Trx presented with increased levels of peritoneal TNF*α* and IL-10 (1.8- and 2.1-fold increase, resp.) and reduced (81%) NO concentrations. ML204 injection in non-SIRS mice administered with Trx resulted in further increase of TNF*α* and IL-10 (Table 2).

## 4. Discussion

Growing evidence has linked the activation of TRPC5 complexes to inflammation [15, 16, 26, 27]. Interestingly, these reports have suggested an anti-inflammatory and immunosuppressive role for these complexes. TRPC5 activation is also involved in multiple organ functions as a protective molecule in diseased states [24, 25]. TRPC5 channels can be activated by a range of endogenous stimuli such as G-protein coupled receptor activation, oxidised phospholipids, H_2_O_2_, and reduced Trx [15, 28–31]. Of note, Trx is involved in the antioxidant machinery of both mammalians and microorganisms. Despite all these evidences, the relevance of TRPC5 complexes to the SIRS associated with bacterial infections has not yet been addressed; moreover, their importance as targets for bacterial Trx has never been explored.

Herein, we evaluated the role of TRPC5 and TRPC4 complexes in a nonlethal mouse model of LPS-induced SIRS and especially its contribution to bacterial Trx signalling in the host. Data gathered from TRPC5^−/−^ mice suggest that the endogenous activation of TRPC5 has little or no participation in the local and systemic inflammatory responses to LPS, as in its absence, only AST levels are regulated. On the other hand, dual blockade of TRPC4/TRPC5 complexes culminated with the regulation of different steps of SIRS (enhanced hypothermia, reduction of peritoneal mononuclear cell numbers, and diminished cytokine release), and this was associated with mortality (15%). These evidences suggest a role for TRPC4 but not TRPC5 receptors in LPS-induced responses. Recent reports demonstrated that LPS does not activate TRPC4 but induces its expression in pulmonary arterial smooth muscle cells *in vitro*; the same studies showed TRPC5 is not expressed on these cells [18, 19]. However, a potential role for TRPC5 in LPS responses is yet to be discarded as little is known of the existence of compensatory mechanisms in TRPC5^−/−^ mice in inflammation. Of note, both TRPC5 deletion and TRPC4/TRPC5 blockade by ML2014 were previously demonstrated to increase cytokine release in complete Freund's adjuvant- (CFA-) injected mice [16], suggesting these channels may respond differently to different inflammatory challenges (i.e., LPS versus CFA).

We also found that in non-SIRS mice, ML204 increases the peritoneal concentrations of TNF*α*, IL-10, and H_2_O_2_ whilst reducing NO levels. Treatment with bacterial Trx resulted in similar responses for TNF*α*, IL-10, and NO, and its coadministration with ML204 further exacerbated the release of TNF*α* and IL-10. These alterations caused by either ML204 or bacterial Trx in non-SIRS animals had little or no effect in the other evaluated parameters, except for body temperature. However, the relevance of these data is unclear, especially in regards to bacterial Trx, as this protein is only expected to be present at systemic levels in the host if there is an ongoing bacterial infection.

Different strains of mice were used in our study (C57BL/6 and 129Si/SvImJ) and, as such, the intensity of the inflammatory response varied between them. Differences between mouse strains have also been shown in other studies in regard to inflammatory mediator release and leukocyte migration [23, 32, 33]. Despite that, we show that *E.coli*-derived Trx regulates a series of inflammatory events in our model, causing reduction of peritoneal leukocytes, cytokines (IL-6 and IL-10), and H_2_O_2_, in addition to impaired macrophage-mediated phagocytosis. These responses were associated with increased mouse mortality (28%). This was expected as the ability of bacterial Trx to mediate mortality during infection is not novel. Indeed, due to its antioxidant properties, bacterial Trx was previously associated with increased mortality by *Salmonella enterica in vivo* [14]. This protein is also known to contribute to bacterial virulence and evasion from the host immune system [1, 2, 10–14].

Our data also shows that cytokine (TNF*α* and IL-6), NO, and H_2_O_2_ release in the peritoneum is exacerbated in SIRS TRPC5^−/−^ mice pretreated with Trx whilst ML204 partially reversed the inhibitory effects of this protein on IL-6 and IL-10 production, without affecting NO and H_2_O_2_ levels. These data allow us to suggest that TRPC4 and TRPC5 channels may play different roles in the generation of inflammatory mediators upon Trx stimuli.

Interestingly, the suppression of macrophage-mediated phagocytosis by Trx was absent in TRPC5^−/−^ and also in those administered with ML204, indicating that both TRPC5 and TRPC4 mediate Trx responses in SIRS mice. Of note, the ability of TRPC4 and TRPC5 to form homo- and heterodimers, in addition to the lack of selective antagonists for each of these receptors, makes difficult to distinguish their individual physiological and pathological roles. Despite that, it was demonstrated herein that these channels may act as additional targets for bacterial Trx during SIRS.

Although different regulatory pathways may be associated with TRPC4 and TRPC5, enhanced mortality (65% rate) was observed in LPS-injected mice treated with *E.coli*-derived Trx and ML204; therefore, suggesting for the first time, a protective role for TRPC4/TRPC5 channels in infections caused by this microorganism. It is possible that TRPC4 and TRPC5 also play similar roles in infections caused by other bacteria, but this remains to be evaluated.

Overall, these data indicate that bacterial Trx effects in LPS-induced responses depend on the activation of both TRPC4 and TRPC5 channels, with these playing distinct and additional roles in disease outcome. Further *in vitro* and *in vivo* studies addressing the potential of TRPC4/TRPC5 selective agonists as protective molecules against bacterial infections remain to be investigated.

## Figures and Tables

**Table 1:** Inflammatory mediator levels in peritoneal lavage samples obtained from TRPC5^+/+^ and TRPC5^−/−^ mice intraperitoneally (i.p.) injected with lipopolysaccharide (LPS) and pretreated subcutaneously with either vehicle (PBS; *n* = 6) or bacterial thioredoxin (Trx; 20 *μ*g/150 *μ*l, twice a day, for 3 days, *n* = 5 − 6). Animals treated with vehicle (PBS) were used as controls (*n* = 4). ^∗^*p* < 0.05 differs from the vehicle + vehicle group; ^#^*p* < 0.05 differs from the vehicle + LPS group; ^&^*p* < 0.05 differs from LPS-injected WT mice pretreated with bacterial Trx.

Inflammatory mediator	TRPC5^+/+^			TRPC5^−/−^		
	Vehicle + vehicle	Vehicle + LPS	Trx + LPS	Vehicle + vehicle	Vehicle + LPS	Trx + LPS
TNF*α* (pg/ml)	47.0 ± 2.7	68.6 ± 5.7	87.3 ± 14.4	49.3 ± 10.3	78.0 ± 8.6	119.0 ± 12.7^∗^
IL-6 (pg/ml)	1339.0 ± 69.0	9822.0 ± 1969.0	7957.0 ± 2141.0	1311.0 ± 38.7	13319.0 ± 4011.0	29657.0 ± 4882.0 ^∗#&^
IL-10 (pg/ml)	254.5 ± 3.3	309.7 ± 12.1	354.2 ± 66.4	274.8 ± 29.2	329.1 ± 19.4	367.0 ± 36.1
NO^x^ (*μ*M)	5.5 ± 1.2	5.3 ± 1.2	9.3 ± 1.1	6.3 ± 0.9	7.5 ± 0.7	27.7 ± 10.7
H_2_O_2_ (*μ*M)	2.5 ± 0.6	4.3 ± 0.8	7.5 ± 0.9	3.1 ± 0.7	3.8 ± 0.8	10.8 ± 4.4

**Table 2:** Inflammatory mediator levels in peritoneal lavage samples obtained from C57BL/6 mice intraperitoneally (i.p.) injected with lipopolysaccharide (LPS) or vehicle (PBS) and pretreated subcutaneously with vehicle (6% DMSO in PBS; *n* = 8), ML204 (1 mg/kg, 150 *μ*l/mice, twice a day, for 6 days; *n* = 5–8), and/or bacterial thioredoxin (Trx, 20 *μ*g/150 *μ*l, twice a day, for 3 days; *n* = 5–8). ^∗^*p* < 0.05 differs from the vehicle + vehicle group; ^#^*p* < 0.05 differs from the vehicle-control group treated with ML204, Trx or ML204 + Trx; ^&^*p* < 0.05 differs from the vehicle + LPS group.

Inflammatory mediator	Vehicle + vehicle	ML204 + vehicle	Trx + vehicle	ML204 + Trx + vehicle	Vehicle + LPS	ML204 + LPS	Trx + LPS	ML204 + Trx + LPS
TNF*α* (pg/ml)	8.8 ± 2.6	15.5 ± 2.5	16.1 ± 2.6	33.2 ± 3.5^∗^	16.3 ± 5.7	14.3 ± 8.1	7.8 ± 3.3	7.3 ± 3.1^#^
IL-6 (pg/ml)	2.1 ± 0.4	2.0 ± 0.7	1.8 ± 0.4	2.9 ± 0.7	403.0 ± 133.1^∗^	144.0 ± 61.1	105.2 ± 55.0^&^	186.9 ± 94.3
IL-10 (pg/ml)	4.8 ± 2.5	12.0 ± 1.4	9.9 ± 1.6	20.7 ± 1.3^∗^	8.4 ± 2.2	1.7 ± 0.8	1.6 ± 0.6	6.3 ± 2.7^#^
NO^x^ (*μ*M)	47.3 ± 7.3	0.1 ± 0.03^∗^	9.0 ± 7.1	6.1 ± 6.0	96.9 ± 10.2^∗^	122.9 ± 24.0^#^	78.2 ± 8.0^#^	81.7 ± 8.4^#^
H_2_O_2_ (*μ*M)	16.5 ± 4.7	30.9 ± 3.4^∗^	19.7 ± 4.6	8.2 ± 3.0	19.4 ± 4.1	19.1 ± 5.2	10.6 ± 2.4	12.3 ± 2.8

## Data Availability

The datasets used to support this study will be made available upon request. Requests should be sent to the corresponding author.
